# Exploring health literacy in Wuhan, China: a cross-sectional analysis

**DOI:** 10.1186/s12889-020-09520-9

**Published:** 2020-09-17

**Authors:** Xin Mei, Qing Zhong, Gong Chen, Yuanxia Huang, Junlin Li

**Affiliations:** 1 Department of Health Education, Wuhan Center for Disease Control and Prevention, No. 288 Machang Road, Changqing Street, Jianghan District, Wuhan, 430024 Hubei China; 2 Medical Department, The Central Hospital of Wuhan, Tongji Medical College, Huazhong University of Science and Technology, No. 26 Shengli Street, Jiang’an District, Wuhan, 430014 Hubei China

## Abstract

**Background:**

In recent years, research on health literacy has become increasingly focused on the health care system and public health. This cross-sectional study aimed to investigate health literacy and analyse the risk factors that affect health literacy in Wuhan, China.

**Methods:**

Multistage stratified random sampling was used to select 5304 urban and rural residents aged 15 to 69 years from 204 monitoring points in 15 districts of Wuhan. Using the Chinese Citizen Health Literacy Questionnaire (HLQ) (2018 edition), a face-to-face survey was conducted from November to December 2018. Risk factors that may affect health literacy were assessed using the Chi-square test and multivariate logistic regression models.

**Results:**

The knowledge rate of health literacy was relatively low (19.3%). The knowledge rate of health-related behaviour and lifestyle (BAL, 17.3%) was the lowest of the three aspects of health literacy, and the knowledge rate of chronic diseases (CD, 19.0%) was the lowest of the six dimensions of health literacy. Respondents who lived in urban areas, had higher education levels, worked as medical staff, had a higher household income and did not suffer from chronic diseases were likely to have higher health literacy.

**Conclusions:**

The health literacy levels of citizens in Wuhan are insufficient and need to improve.

## Background

Health literacy [1–3] is often defined as the ability to obtain, process, and understand basic health information and services to make appropriate health decisions. As a variable associated with health outcomes, health literacy is an important component of people’s health behaviours, health quality, and access to health information and health care [4]. Lower health literacy leads to obstacles in communication between health-care professionals and patients, obstruction of citizens’ access to health information and inefficiency in self-health management [5–7]. Therefore, health literacy should be an important priority for the government and researchers [8]. Because of the critical role of health literacy in health quality, many countries, such as the United States, Canada and Australia, have treated it as a national health indicator [9–12].

In January 2008, the Chinese Ministry of Health finalized the bulletin “Chinese Resident Health Literacy—Basic Knowledge and Skills (Trial)” [13, 14], which was the first government document to define citizens’ health literacy and played a vital role in the development and promotion of health education in China [15]. According to the bulletin, a Delphi process was conducted among relevant experts to choose indexes for the Chinese Citizen Health Literacy Questionnaire (HLQ) to be administered to Chinese residents [16]. After the HLQ was determined to display strong construct validity, reliability, and high acceptability [17], the national health literacy survey was conducted in 2012 and 2016. Health literacy knowledge rates, which are used to measure the level of health literacy, refer to the proportion of people with good knowledge of health literacy in the total population. The results indicated that the health literacy knowledge rates in 2012 and 2016 were 8.80 and 11.58% [18, 19], respectively. Widespread surveys of health literacy have been conducted in China, and the overall level of health literacy is low and a large difference in the levels of health literacy of rural and urban residents.

Health literacy is an important determinant of health. It is a comprehensive reflection of economic and social development and is influenced by various factors, such as politics, economics, culture, education and the health development level [20]. With the acceleration of urbanization in China, areas of central China around Wuhan will become the most rapidly developing district in the future. Wuhan, the capital of Hubei Province and a core city in central China, is an important industrial base, science and education centre and comprehensive transportation hub, placing it at the “heart” of Chinese economic geography [21]. With the rapid development of the economy, people’s living conditions have improved substantially, and interest in health is growing. Therefore, studies investigating health literacy have important practical value. The objectives of this study are to 1) investigate the health literacy level of citizens in Wuhan, China and 2) analyse the risk factors that may affect health literacy.

## Methods

### Participants

In all, 5304 citizens aged 15 to 69 years who had lived in the sampled regions for more than 6 of the previous 12 months were selected in Wuhan, and 5205 were interviewed from November to December 2018 in this cross-sectional survey. Participants were excluded from the survey if they refused to participate or were unable to communicate. The questionnaire was self-administered. However, if participants were unable to complete the questionnaire due to impaired vision, a poor understanding, a lower education level or other similar reasons, an interview was conducted as an alternative. In this case, the investigators would complete the questions in a neutral manner on behalf of the participants. An incentive was provided to encourage them to participate in the survey, and participants were sent small gifts (20 packages of toilet paper) as a reward for participating. The response rate of the questionnaire was 98.1%. A multistage stratified random sampling method [22] was used to recruit participants. The sampling method was divided into three stages. First, residential committee villages or administrative villages were regarded as primary sampling units (PSUs), and 204 monitoring points (residential committee villages or administrative villages) were selected from the whole city using the probability-proportional-to-size sampling (PPS) method. Then, 26 households were randomly selected from each chosen monitoring point. Finally, one eligible resident from each household was selected randomly using a Kish selection table [23]. This study was approved by the Ethics Committee of Wuhan Centre for Disease Control and Prevention, China. Written informed consent was obtained from all participants.

### Questionnaire

Data were obtained in face-to-face interviews with the HLQ (2018 edition) [16] developed by the Chinese Ministry of Health. As Chinese is the common and official language of China, the questionnaire was prepared in simplified Chinese. The questionnaire consisted of three parts: (1) family support questionnaire, (2) basic personal situation, and (3) health literacy content. Based on the “Chinese Resident Health Literacy—Basic Knowledge and Skills (Trial)” and existing public health issues in China, the health literacy section (50 questions) was further categorized into three aspects and six dimensions. The three aspects were (1) knowledge and attitudes (KAA, 22 questions), (2) health-related behaviour and lifestyle (BAL, 16 questions), and (3) health-related skills (HRS, 12 questions). The six dimensions were (1) scientific views of health (SVH, 8 questions), (2) infectious diseases (ID, 6 questions), (3) chronic diseases (CD, 9 questions), (4) safety and first aid (SAFA, 10 questions), (5) medical care (MC, 11 questions), and (6) health information (HI, 6 questions). An overall health literacy score was computed as the sum of all three aspects and six dimensions.

### Evaluation method

Four types of questions were included in the scale: true-or-false questions, single-answer questions, multiple-answer questions, and situation questions. For true-or-false questions and single-answer questions, 1 point was assigned to the correct answer. For multiple-answer questions, 2 points were assigned when all of the correct answers and no incorrect answers were chosen. For situation questions, the participants were required to answer single- or multiple-answer questions after reading the given material. A score of 0 points was recorded for a wrong answer. The overall health literacy score ranged from 0 to 66 points. The total points scored in the three aspects KAA, BAL, and HRS were 28, 22 and 16, respectively, and the total points scored in the six dimensions SVH, ID, CD, SAFA, MC, and HI were 11, 7, 12, 14, 14, and 8, respectively.

### Variables

The participants were divided into 2 categories: (1) poor knowledge of health literacy (total health literacy score < 80% of the overall score, with a total score < 53 points) and (2) good knowledge of health literacy (total health literacy score ≥ 80% of the overall score, with a total score ≥ 53 points) [4, 24]. The knowledge rate (%) of health literacy was calculated using the following formula: total number of participants with good knowledge of health literacy / total number surveyed × 100%. The knowledge rates of the three aspects and six dimensions of health literacy were calculated similarly. Quality control was applied to the whole investigative process. Based on previous studies [25–30] and our results from the Chi-square test, the possible risk factors that may affect health literacy were chosen. The possible risk factors considered were the area of residence (0 = rural and 1 = urban), age in years (1 = 15–24, 2 = 25–34, 3 = 35–44, 4 = 45–54, 5 = 55–64, and 6 = 65–69), education (1 = illiterate, 2 = primary school, 3 = junior school, 4 = high school, 5 = college, and 6 = master’s degree or higher), occupation (1 = civil servant, 2 = teacher, 3 = medical staff, 4 = staff at other public institutions, 5 = student, 6 = farmer, 7 = worker, 8 = staff of other enterprises, and 9 = other), number of people in the household (1 = 1–3, 2 = 4–6, and 3 = ≥7), average annual household income (CNY) (1 = < 30,000, 2 = 30,000-50,000, 3 = 50,000-100,000, 4 = 100,000-300,000, and 5 = ≥300,000), suffering from chronic diseases (0 = no and 1 = yes), and self-reported health status (1 = excellent, 2 = good, 3 = average, 4 = relatively poor, and 5 = poor).

### Statistical analysis

The data were meticulously sorted, cleaned, and analysed with SPSS version 21 (International Business Machines Corporation, Armonk, NY, USA). A descriptive analysis (frequencies, percentages, and means with standard deviations) of participant characteristics was performed. The Chi-square test was used to compare the knowledge rates of health literacy among subgroups. A multivariate logistic regression analysis was conducted to assess the associations of multiple potential risk factors with the knowledge rate of health literacy. The multivariate logistic regression analysis was used to adjust for the risk factors associated with health literacy. Statistical significance was defined as a *p*-value < 0.05 (two-sided).

## Results

### Sociodemographic characteristics of the participants

As shown in Table 1, 2408 of the participants (46.3%) were male and 2797 (53.7%) were female. More than half of the participants (63.7%) lived in urban areas. The average age was 49.1 ± 13.7 years. Most participants were educated at the junior high school, high school or college level, accounting for 84.0% of participants. Approximately 13.4% of the participants were farmers, and the percentage of participants with 1–3 people in the household was 71.0%. More than 60% of the participants had an average annual income of no more than 100,000 CNY. Of the participants, 50.1% had chronic diseases, and over 60% reported that they were in excellent or good health.

**Table 1:** Sociodemographic characteristics and knowledge rate of health literacy in Wuhan in 2018

Characteristics	N (%) ^a^	> 80% score ^b^	Knowledge rate (%)	χ^2^	*P*
Area of residence					
Urban	3315 (63.7)	773	23.3	95.20	< 0.001
Rural	1890 (36.3)	231	12.2		
Gender					
Male	2408 (46.3)	450	18.7	1.04	0.308
Female	2797 (53.7)	554	19.8		
Age, years					
15–24	209 (4.0)	65	31.1	210.58	< 0.001
25–34	759 (14.6)	212	27.9		
35–44	853 (16.4)	261	30.6		
45–54	1232 (23.7)	218	17.7		
55–64	1343 (25.8)	154	11.5		
65–69	809 (15.5)	94	11.6		
Ethnicity					
Han	5168 (99.3)	993	19.2	2.61	0.106
Other	37 (0.7)	11	29.7		
Education					
Illiterate	141 (2.7)	7	5.0	768.04	< 0.001
Primary school	550 (10.6)	30	5.5		
Junior school	1431 (27.5)	87	6.1		
High school	1524 (29.3)	235	15.4		
College	1417 (27.2)	567	40.0		
Master’s degree or higher	142 (2.7)	78	54.9		
Occupation					
Civil servant	89 (1.7)	39	43.8	677.64	< 0.001
Teacher	231 (4.4)	130	56.3		
Medical staff	127 (2.4)	71	55.9		
Staff at other public institutions	608 (11.7)	228	37.5		
Student	110 (2.1)	43	39.1		
Farmer	699 (13.4)	41	5.9		
Worker	684 (13.1)	56	8.2		
Staff of other enterprises	815 (15.7)	144	17.7		
Other	1842 (35.4)	252	13.7		
Number of people in the household					
1–3	3695 (71.0)	680	18.4	6.450	0.040
4–6	1447 (27.8)	310	21.4		
≥ 7	63 (1.2)	14	22.2		
Average annual household income, CNY					
< 30,000	539 (10.4)	21	3.9	268.12	< 0.001
30,000-50,000	969 (18.6)	87	9.0		
50,000-100,000	1826 (35.1)	367	20.1		
100,000-300,000	1589 (30.5)	478	30.1		
≥ 300,000	282 (5.4)	51	18.1		
Native population					
Yes	4648 (89.3)	894	19.2	0.09	0.771
No	557 (10.7)	110	19.7		
Suffering from chronic diseases ^c^					
Yes	2610 (50.1)	370	14.2	87.91	< 0.001
No	2595 (49.9)	634	24.4		
Self-reported health status					
Excellent	1138 (21.9)	238	20.9	9.66	0.047
Good	2203 (42.3)	444	20.2		
Average	1643 (31.6)	288	17.5		
Relatively poor	186 (3.6)	31	16.7		
Poor	35 (0.7)	3	8.6		
Total	5205 (100.0)	1004	19.3	–	–

^a^Percentage of all participants; ^b^number of participants with a total score greater than 80%; ^c^ chronic diseases included hypertension, heart disease, cerebrovascular disease (stroke, cerebral infarction, cerebral thrombosis), diabetes, and malignant cancer

### The association between the knowledge rate of health literacy and the sociodemographic characteristics

As indicated in Table 1, there were significant differences in the knowledge rates of health literacy among by area of residence, age, education, occupation, number of people in the household, household income, chronic diseases and health status (*p* < 0.05) but not by gender, ethnicity and native population.

### Average score, knowledge rate of total health literacy and the three aspects and six dimensions of health literacy

The average scores for each health literacy scale are shown in Table 2. The knowledge rates of the three aspects KAA, HRS, and BAL were 33.9, 33.2 and 17.3%, respectively. Additionally, for the six dimensions, the knowledge rate in descending order was 57.8, 52.8, 41.0, 27.8, 26.1, and 19.0% for SAFA, SVH, HI, MC, ID, and CD, respectively.

**Table 2:** Average score and knowledge rate of each scale in the three aspects and six dimensions

Variables ^a^	Number of questions	Total points	80% of the total score	Mean ± SD	> 80% score ^b^	Knowledge rate (%)
Three aspects						
KAA	22	28	22.4	19.1 ± 5.0	1767	33.9
BAL	16	22	17.6	13.3 ± 4.5	900	17.3
HRS	12	16	12.8	10.5 ± 3.6	1728	33.2
Six dimensions						
SVH	8	11	8.8	7.4 ± 2.5	2749	52.8
ID	6	7	5.6	4.6 ± 1.5	1356	26.1
CD	9	12	9.6	7.0 ± 2.7	988	19.0
SAFA	10	14	11.2	10.5 ± 3.0	3008	57.8
MC	11	14	11.2	8.7 ± 2.8	1449	27.8
HI	6	8	6.4	4.8 ± 2.1	2132	41.0
Health literacy	50	66	52.8	42.9 ± 11.7	1004	19.3

^a^*KAA* Knowledge and attitudes, *BAL* Health-related behaviour and lifestyle, *HRS* Health-related skills, *SVH* Scientific views of health, *ID* Infectious diseases, *CD* Chronic diseases, *SAFA* Safety and first aid, *MC* Medical care, *HI* Health information. ^b^ Number of participants with a total score > 80%

### Multivariate logistic regression analysis of risk factors associated with the health literacy knowledge rate

The variables with statistical significance in the Chi-square test (Table 1) were examined in the multivariate logistic regression analysis. As shown in Table 3, participants living in urban areas (OR = 1.31, 95% CI: 1.08, 1.60) were significantly likely to have a higher knowledge rate of health literacy than participants living in rural areas. Compared to participants who were illiterate, participants with an education level of high school (OR = 2.27, 95% CI: 1.00, 5.13), college (OR = 5.36, 95% CI: 2.35, 12.23) and master’s degree or higher (OR = 6.67, 95% CI: 2.70, 16.46) were likely to have a significantly higher health literacy knowledge rate. Medical staff (OR = 2.42, 95% CI: 1.36, 4.32) were likely to have higher health literacy levels than residents whose occupation was a civil servant. As the average household income increased, the participants were more likely to present higher health literacy levels. Participants suffering from chronic disease were more likely to present lower health literacy levels. Of these factors, living in an urban area, education level (except primary school and junior school), working as medical staff and having a higher annual household income were positively correlated with health literacy, while being a worker or suffering from chronic diseases were negatively correlated with health literacy.

**Table 3:** Multivariate logistic regression analysis of risk factors associated with health literacy in participants stratified by different socio-demographic characteristics

Parameters	B	S.E.	Wald	df	P	OR	OR 95% CI
Residence							
Rural	ref						
Urban	0.27	0.10	7.26	1	0.01	1.31	(1.08, 1.60)
Age, years old							
15–24	ref						
25–34	0.06	0.24	0.07	1	0.79	1.07	(0.67, 1.70)
35–44	0.29	0.24	1.46	1	0.23	1.34	(0.83, 2.14)
45–54	0.01	0.24	0.00	1	0.96	1.01	(0.63, 1.63)
55–64	− 0.37	0.25	2.29	1	0.13	0.69	(0.42, 1.12)
65–69	− 0.01	0.26	0.00	1	0.96	0.99	(0.59, 1.66)
Education							
Illiterate	ref						
Primary school	0.02	0.44	0.00	1	0.96	1.02	(0.43, 2.41)
Junior school	0.03	0.42	0.00	1	0.95	1.03	(0.45, 2.32)
High school	0.82	0.42	3.88	1	< 0.05	2.27	(1.00, 5.13)
College	1.68	0.42	15.97	1	< 0.01	5.36	(2.35, 12.23)
Master’s degree or higher	1.90	0.46	16.91	1	< 0.01	6.67	(2.70, 16.46)
Occupation							
Civil servant	ref						
Teacher	0.60	0.26	5.19	1	0.02	1.83	(1.09, 3.07)
Medical staff	0.88	0.29	9.02	1	< 0.01	2.42	(1.36, 4.32)
Staff at other public institutions	0.12	0.24	0.24	1	0.62	1.13	(0.70, 1.81)
Student	0.31	0.37	0.72	1	0.40	1.37	(0.67, 2.80)
Farmer	−0.28	0.31	0.85	1	0.36	0.75	(0.41, 1.38)
Worker	−0.79	0.28	8.10	1	< 0.01	0.46	(0.27, 0.78)
Staff of other enterprises	−0.72	0.25	8.58	1	< 0.01	0.49	(0.30, 0.79)
Other	−0.45	0.24	3.49	1	0.06	0.64	(0.40, 1.02)
Number of people in the household							
1–3	ref						
4–6	−0.04	0.09	0.19	1	0.67	0.96	(0.81, 1.15)
≥ 7	0.37	0.35	1.11	1	0.29	1.44	(0.73, 2.86)
Average household income per month (CNY)							
< 30,000	ref						
30,000–50,000	0.51	0.26	3.92	1	< 0.05	1.67	(1.01, 2.76)
50,000–100,000	1.21	0.24	25.81	1	< 0.01	3.36	(2.11, 5.37)
100,000–300,000	1.41	0.24	34.73	1	< 0.01	4.11	(2.57, 6.57)
≥ 300,000	0.65	0.29	5.06	1	0.02	1.91	(1.09, 3.35)
Suffering from chronic diseases ^a^							
No	ref						
Yes	−0.18	0.09	4.41	1	0.04	0.83	(0.70, 0.99)
Self-reported health status							
Excellent	ref						
Good	0.05	0.10	0.26	1	0.61	1.05	(0.86, 1.29)
General	0.05	0.11	0.17	1	0.68	1.05	(0.84, 1.31)
Relatively poor	0.20	0.24	0.66	1	0.42	1.22	(0.76, 1.96)
Poor	−0.11	0.65	0.03	1	0.87	0.90	(0.25, 3.23)
Constant	−3.34	0.58	33.20	1	< 0.01	0.04	–

^a^Chronic diseases included hypertension, heart disease, cerebrovascular disease (stroke, cerebral infarction, cerebral thrombosis), diabetes, and malignant cancer

## Discussion

The analysis of the risk factors may not only help identify key groups and areas where health literacy is limited but also indicate targeted intervention measures. With the development of the economy, we should strengthen quality-oriented education, popularize health-related knowledge and skills, and strive to improve people’s health literacy levels. In our study conducted in Wuhan, China, the knowledge rate of health literacy was 19.3% (with an average score of 42.9 ± 11.7), which was a little higher than the average level of Chinese residents in 2018 (17.1%) [31], but still constituted a large gap compared with the knowledge rate in developed areas [17] (32.3% in Beijing, China). The high proportions of people with a limited knowledge rate of health literacy indicate that the health literacy deficit is a challenge for researchers, community organizations, health care providers, and policy-makers [17].

In terms of the three aspects, the knowledge rate of health literacy related to BAL (17.3%) was comparatively lower than the other two components of comprehensive health literacy: KAA (33.9%) and HRS (33.2%). Based on this finding, Chinese people may lack awareness that a healthy lifestyle can help prevent diseases rather than only cure them. Because China is a developing country, this lack of awareness may be associated with socio-cultural and economic limitations. In addition, based on the theory of knowledge, attitude and practice (KAP), changes in health-related behaviour are divided into three continuous processes: obtaining knowledge, forming faith and producing behaviour. Knowledge is comparatively easy to obtain, but the formation of faith and subsequent production of health-related behaviour takes longer, indicating that health education and promotion should focus not only on health knowledge but also on health-related behaviour. Risk factors may affect the health-related behaviour of different groups in different ways; thus, individually targeted health education and health promotion activities should be developed.

The knowledge rate of CD (19.0%) was the lowest of the 6 dimensions, consistent with the study by Zhang Y et al. [32]. Chronic disease is the leading global health threat, and the costs of chronic disease are steep, not only for individuals but also for families and society [33]. According to the survey, 29.08% of the participants suffered from chronic diseases, and a lower health literacy knowledge rate of CD was observed. Participants who were not suffering from chronic diseases had a higher knowledge rate of health literacy, consistent with the results reported by Rafferty AP et al. [34] and Beauchamp A et al. [35]. Restricted interpersonal relations, social resources and scope of activity might be related to the accessibility of health information, which may affect the level of health literacy. Inadequate health literacy poses a major barrier to educating patients with chronic diseases [35]. Meanwhile, citizens with low CD levels may have difficulty improving their lifestyles, managing their diseases, and participating in complex decisions about treatment [8]. Hence, we should strengthen health education and promote health literacy in the future, particularly in terms of CD.

In our study, the multivariate logistic regression analysis indicated that the area of residence, education level, occupation, household income and suffering from chronic diseases may affect health literacy.

A higher knowledge rate of health literacy was observed for participants who lived in urban areas than participants who lived in rural areas, consistent with the results reported by Wang W et al. [36]. Thus, a higher health literacy level was associated with an urban residence. The potential explanation is that participants living in urban areas have easy access to high-quality health information and medical information services. Therefore, rural areas are key areas for future health education and health promotion.

The education level may be one of the most important positive factors contributing to health literacy [37]. The knowledge rate of health literacy increased as the education level increased (except between primary school and junior school), consistent with the results reported by Manafo E et al. [38] and McClintock H et al. [39]. The same result was obtained for the factor of occupation, as medical staff have a higher level of health literacy, which might result from medical professional knowledge and a higher education level [15]. A low level of education is related to a poor reading and comprehension ability, which may cause people to make poor or indirect use of health information and media. Therefore, improving the education level of residents is fundamental to increasing health literacy. For people with a low educational level, we should apply a popular propagation mode and develop health communication materials that are easy to understand.

Higher health literacy levels have consistently been reported to be associated with higher household income [24, 26], similar to our results. The knowledge rate of health literacy was lower in participants with a lower household income level. With increasing household income, the participants likely paid more attention to self-health management and quality of life. According to a study by Pawlak R [40] a lower income level is an important reason for a low health literacy level, which leads to worse health and a greater rate of hospitalization. For low-income groups, an increase in the household income and improvements in the health care system are very important for improving the health level.

In our study, the health literacy of people with chronic diseases was low, consistent with the results reported by Sorensen K et al. [9] and Wu L et al. [41]. Notably, the risk of chronic diseases in elderly people also increases when they are suffering from chronic diseases. Therefore, interventions designed to help people suffering from chronic diseases stay healthy and improve the quality of life in their later years are very important [22, 42].

In conclusion, the survey extends the scientific evidence on health literacy by measuring the knowledge rate of health literacy in Wuhan, China. Limited health literacy and a social gradient in health literacy represent important challenges for health policies and practices in Wuhan. This health literacy deficit and inequality should be addressed by Wuhan health planners and policy-makers, and appropriate public health and health promotion strategies should be developed.

This study has some limitations. All the data were analysed based on self-report measures. Therefore, the programme may be subject to bias. First, the study employed a cross-sectional design; hence, the analysis did not permit causal inferences. Second, health literacy, by definition, emphasizes health skills and applications. However, the survey of health literacy focused on knowledge, which has little involvement in skills and application. Finally, we assessed health literacy in three categories: total health literacy, three aspects of health literacy and six dimensions of health literacy. However, when designing the questionnaire, the complexity and quantity of questions in different categories should be balanced.

## Conclusions

The knowledge rate of health literacy of Wuhan residents is relatively low (19.3%). The knowledge rate of health-related behaviour and lifestyle (BAL, 17.3%) is the lowest of the three aspects of health literacy, and the knowledge rate of chronic diseases (CD, 19.0%) is the lowest of the six dimensions of health literacy. The knowledge rate of health literacy was negatively associated with respondents living in rural areas, with a low education level, a low household income, and suffering from chronic diseases. Thus, health education and promotion interventions should be targeted to high-priority topics (BAL and CD), key areas (rural) and high-risk populations (people who are illiterate, have a low annual income and suffer from chronic diseases) to improve health outcomes.

## Data Availability

The data used and/or analysed in the current study are not publicly available because restrictions apply to the availability of these data. Data are, however, available from the corresponding author on reasonable request and with permission of the Wuhan Center for Disease Control and Prevention.

## Abbreviations

HLQ: Chinese Citizen Health Literacy Questionnaire
KAA: Knowledge and attitudes
BAL: Health-related behaviour and lifestyle
HRS: Health-related skills
SVH: Scientific views of health
ID: Infectious diseases
CD: Chronic diseases
SAFA: Safety and first aid
MC: Medical care
HI: Health information
